# Rate control treatment with calcium channel blockers and beta blockers for patients with atrial fibrillation: a systematic review and meta-analysis

**DOI:** 10.1093/ehjopen/oeag062

**Published:** 2026-04-16

**Authors:** Tim Koldenhof, Barzi Gareb, Marcelle D Smit, Thijmen S A Bergwerff, Michiel Rienstra, Robert G Tieleman

**Affiliations:** Department of Cardiology, Martini Hospital, Van Swietenplein 1, Groningen 9728 NT, The Netherlands; Department of Cardiology, University of Groningen, University Medical Center Groningen, Hanzeplein 1, Groningen 9713 GZ, The Netherlands; Department of Oral and Maxillofacial Surgery, University Medical Center Groningen, Hanzeplein 1, Groningen 9713 GZ, Groningen, The Netherlands; Department of Cardiology, Martini Hospital, Van Swietenplein 1, Groningen 9728 NT, The Netherlands; Department of Cardiology, University of Groningen, University Medical Center Groningen, Hanzeplein 1, Groningen 9713 GZ, The Netherlands; Department of Cardiology, University of Groningen, University Medical Center Groningen, Hanzeplein 1, Groningen 9713 GZ, The Netherlands; Department of Cardiology, Martini Hospital, Van Swietenplein 1, Groningen 9728 NT, The Netherlands; Department of Cardiology, University of Groningen, University Medical Center Groningen, Hanzeplein 1, Groningen 9713 GZ, The Netherlands

**Keywords:** Atrial fibrillation, Rate control, Calcium channel blocker, Beta blocker

## Abstract

**Aims:**

Controlling heart rate is a key objective in atrial fibrillation (AF) management, commonly achieved with beta blockers or non-dihydropyridine calcium channel blockers, both class I guideline recommendations. However, their long-term effectiveness has rarely been compared. This review aimed to critically evaluate all available evidence comparing these drug classes in AF treatment.

**Methods and results:**

We systematically searched MEDLINE, Embase, Web of Science, and Cochrane Central for original studies published up to 1 March 2025. Eligible studies included AF patients, compared calcium channel blockers and beta blockers, and reported heart rate or other clinically relevant outcomes. The primary outcome was 24-hour heart rate on Holter monitoring. Meta-analyses were performed where data were sufficiently homogeneous. Of 1906 identified records, 25 met inclusion criteria. Five studies reported 24-h mean heart rate, showing no difference between drug classes (mean difference: 3.6 b.p.m., 95% CI: −2.9 to 10.1, *P* = 0.274). Eight studies reported maximum exercise heart rate, which was significantly higher with calcium channel blockers than with beta blockers (mean difference: 10.5 b.p.m., 95% CI: 3.3 to 17.7, *P* = 0.043). Other outcomes were too heterogeneous for meta-analysis, though three studies reported increased peak oxygen uptake with calcium channel blockers.

**Conclusion:**

Calcium channel blockers and beta blockers provide comparable control of mean heart rate in AF. However, patients on calcium channel blockers have a higher maximum heart rate during exercise. In our qualitative synthesis, some studies reported higher peak oxygen uptake calcium channel blocker treated patients, these results should be seen as hypothesis generating.

## Graphical Abstract

**Graphical Abstract:**
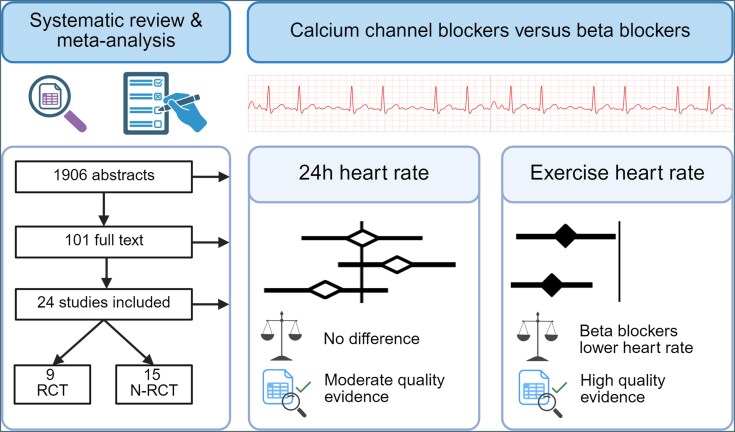

What’s new

- During atrial fibrillation calcium channel blockers and beta blockers are equally effective at reducing heart rate measured on 24 h Holter monitoring.
- During atrial fibrillation calcium channel blockers allow for higher heart rates during peak exercise, potentially enabling better exercise capacity and peak oxygen uptake.

## Introduction

Ventricular rate control is an instrumental part of atrial fibrillation (AF) management and is recommended by the European society of cardiology guidelines in all patients with AF, even as background therapy during rhythm control therapy.^1^ The aim of rate control is to reduce the ventricular heart rate during AF, reduce symptoms and improve quality of life, improve haemodynamics, prevent heart failure and reduce cardiovascular morbidity.^2^

Both beta blockers and non-dihydropyridine calcium channel blockers are given a class I recommendation with level of evidence B as first line rate control dugs in the latest European guidelines.^1^ In the absence of heart failure with reduced ejection fraction or severe obstructive pulmonary disease no preference is given in the guidelines. Large clinical trials comparing calcium channels and beta blockers for long-term rate control in AF are lacking. The comparative studies that have been performed are heterogeneous in terms of sample size, follow up duration and outcomes, and therefore fail to provide a uniform rate control drug recommendation.^3–5^

The aim of this systematic review is to compare the available evidence on the effects of calcium channel blockers and beta blockers on heart rate during 24 h Holter monitoring during AF to guide evidence-based clinical decisions.

## Methods

This systematic review was reported following the Preferred Reporting Items for Systematic Review and Meta-Analyses guidelines^6^ and recorded in the International Prospective Register of Systematic Reviews (PROSPERO) under registration number CRD42024526695. The full methods of this systematic review have previously been published.^7^

### Eligibility criteria

The research question is formulated using the Population, Intervention, Comparison, Outcomes, Study design (PICOS) approach. The PICOS elements are displayed in *Table 1*. The population (P) included patients, over 18 years of age with AF. The intervention (I) group was treated with oral non-dihydropyridine calcium channel blockers, verapamil or diltiazem. The control group was treated with oral beta blockers. The primary outcome (O) was 24 h heart rate during Holter monitoring. Secondary outcomes were maximum heart rate during exercise test, heart rate during rest, major adverse cardiac and cerebrovascular event, all cause morality, cardiovascular mortality, stroke, new onset or worsening heart failure, heart rate during sinus rhythm, sinus bradycardia, progression of AF, need for cardioversion, need for AF ablation, emergency department visits for AF, need for cardiovascular hospitalizations, quality of life, symptoms of AF, exercise tolerance and drug side effects. There were no language restrictions for inclusion. The included study types (S) were randomized controlled trials (RCT), including cluster RCTs, controlled, (non-randomized) clinical studies and retrospective and prospective cohort studies and case series. For articles in any language other than English, Dutch or German, the abstract and full text article were translated to English.

**Table 1:** Population, intervention, comparison and outcomes

PICO element	Criteria
P: population	Adults over the age of 18 with atrial fibrillation
I: intervention	Oral non-dihydropyridine calcium channel blockers, verapamil or diltiazem
C: comparison	Oral beta blockers
O: outcome	Primary: heart rate in AF during 24 h Holter monitoring Secondary: maximum heart rate during exercise test, heart rate during rest, major adverse cardiac and cerebrovascular event (MACCE), all cause morality, cardiovascular mortality, stroke, new onset or worsening heart failure, heart rate during sinus rhythm, sinus bradycardia, progression of AF, need for cardioversion, need for AF ablation, emergency department visits for AF, need for cardiovascular hospitalizations, quality of life, symptoms of AF, exercise tolerance and drug side effects.
S: studies	Randomized controlled trials, including cluster RCTs, controlled, (non-randomized) clinical studies and retrospective and prospective cohort studies and case series.

Although retrospective studies, cohort studies and case series would most likely not be suitable for inclusion in the meta-analyses, we opted to include them in the search because they may add novel insights for the narrative synthesis and provide a foundation for future research.

### Exclusion criteria

Studies were excluded if: (1) the majority (i.e. ≥50%) of patients had AF induced by inflammation, hyperthyroidism, or surgery; (2) patients were treated with intravenous non-dihydropyridine calcium channel blockers or intravenous beta blockers; (3) the majority of patients in the beta blocker arm were treated with sotalol; (4) the manuscripts were not peer-reviewed; or (5) the studies were animal studies, treatment guidelines, or opinion papers.

### Search strategy

A comprehensive search was conducted in MEDLINE, Embase, Web of Science and Cochrane CENTRAL from inception to 1 March 2025, (see Supplementary files Supplementary material online, *Tables S1*–*S4*). In addition, references lists of included articles were screened and AF experts (M.R., R.G.T., M.D.S.) were asked for any relevant studies missing. No language or date restrictions were applied.

### Study selection

The first 50 titles and abstracts were screened by three researchers (T.K., R.G.T., M.R.) for eligibility to reach consensus. The remaining abstracts were screened according to the inclusion and exclusion criteria by two separate researchers (T.K. or T.S.A.B. and one of the following reviewers: R.G.T., M.R., M.D.S.). If an abstract was not available, the full text was assessed in the next round. All included references were subsequently assessed in full text by two separate researchers (T.K. or T.S.A.B. and one of the following reviewers: R.G.T., M.R., M.D.S.). Conflicts, during abstract and full text screening, were resolved through discussion. If unsuccessful, a third reviewer made the final decision. For both abstract and full text screening, the agreement between the two reviewers was assessed by calculating the percentage of agreement as well as the Cohen’s Kappa.

### Data collection

Data were extracted using a pre-defined form (*Table 2*) by one researched (T.K.) and verified by a second reviewer (M.R.). Uncertainties were discussed (T.K., M.R., R.G.T.) until consensus was reached. In some studies, there were four treatment arms: two involving calcium channel blockers and two involving beta blockers. For the purposes of the meta-analyses, we combined the calcium channel blocker arms into a single group, and did the same for the beta blocker arms, to avoid multiple statistical comparisons following the appropriate methods.^17^

**Table 2:** Datasets extracted for included studies in meta analyses

Study	Study design	Year publication	Number of patients with calcium channel blockers	Number of patients with beta blockers	Calcium channel blocker mean heart rate	Calcium channel blocker heart rate SD	Beta blocker mean heart rate	Beta blocker heart rate SD	With digoxin
**Datasets extracted for studies included in 24 h mean heart rate meta-analysis**									
Wang *et al*. (1980)^8^	Non-randomized open label crossover trial	1980	8	8	101.3	26.7	89.4	18.2	No
Koh *et al*. (1995)^9,10^	Randomized open label crossover trial	1995	35	35	73	4	64	4	Yes
Farshi *et al*. (1999)^11a^	Randomized open label crossover trial	1999	12	12	80	15.5	75.9	11.7	No
Farshi *et al*. + digoxin (1999)^11a^	Randomized open label crossover trial	1999	12	12	67.3	14.1	65	9.4	Yes
Tsuneda *et al*. (2006)^12^	Multicentre randomized controlled (open label) trial with partial crossover	2006	22	19	83	14	78	12	No
Ulimoen *et al*. (2013)^3^,^b^	Randomized, investigator-blind, crossover trial	2013	60	60	78	11	83	11	No
**Datasets extracted for studies included in max heart rate during exercise meta-analysis**									
Myers *et al*. (1987)^13^	Partially-randomized open label crossover trial	1987	9	9	142	27	118	20	No
Matsuda *et al*. (1991)^14^	Randomized open label crossover trial	1991	10	10	138	28	138	28	No
Dahlstrom *et al*. (1992)^15^	Randomized double-blind crossover trial	1992	13	13	164	29	163	20	Yes
Koh *et al*. (1995)^9,10^	Randomized open label crossover trial	1995	35	35	154	5	135	5	Yes
Farshi *et al*. (1999)^11a^	Randomized open label crossover trial	1999	12	12	151	27	130	34	No
Farshi *et al*. + digoxin (1999)^11^ ^a^	Randomized open label crossover trial	1999	12	12	146	40	126	29	Yes
Tsuneda *et al*. (2006)^12^	Multicentre randomized controlled (open label) trial with partial crossover	2006	22	19	167	30	152	27	No
Ulimoen *et al*. (2013)^3b^	Randomized, investigator-blind, crossover trial	2013	60	60	158	29	156	30	No
Enge *et al*. (2024)^16c^	Randomized, investigator-blind	2024	49	44	160	25.5	156.5	26	No

SD, standard deviation.

^a^Separate groups with and without digoxin combined into a single variables for main analysis, separate variables used for with/without digoxin sub-analyses.

^b^Separate groups of verapamil and diltiazem combined and metoprolol and carvedilol combined.

^c^Repeated measurements on two consecutive days were combined into singular outcome data.

### Risk of bias assessment

Risk of bias was assessed using the Cochrane risk-of-bias tool for randomized trials (RoB 2) for randomized studies and the Risk Of Bias In Non-Randomized Studies—of Interventions for non-randomized studies. Risk of bias was independently assessed by two out of four reviewers (TK, MDS, RGT, MR). Conflicting outcomes were resolved through discussion or majority decision by a third reviewer.

### Statistical analysis

Meta-analyses were performed comparing mean heart rate and maximum heart rate during 24 h Holter monitoring and mean and maximum heart rate during exercise test. Assessing comparability and assessing clinical diversity between trials in the meta-analyses was performed using the Clinical Diversity In Meta-analyses (CDIM) tool.^18^ When clinical diversity was high (CDIM score >18), and high diversity domains could not be addressed in subgroup analyses, meta-analysis was not performed due to substantial clinical heterogeneity. For continuous outcomes, the sample size, means, and corresponding standard deviations were used to estimate mean differences (MD) or, when the same outcome was measured in various ways, standardized mean differences (SMD) with corresponding 95% confidence and prediction intervals. For binary outcomes, the sample sizes and number of events were used to estimate odds ratios with corresponding 95% confidence and prediction intervals. Statistical heterogeneity between studies was assessed using the *I*^2^ statistic along with the corresponding χ^2^ test. A sensitivity analysis for the primary outcome using the restricted maximum likelihood model to estimate the between-study variance was be conducted. The pre-defined subgroup analysis comparing calcium channel blockers vs. beta blockers with and without co-treatment with digoxin was performed^17^: Sensitivity analyses were performed excluding studies with a high risk of bias and omitting one study at a time (i.e. influence analysis). Reporting bias was assessed using funnel plots if ≥10 studies were included and statistical heterogeneity was absent (*I*^2^ < 50%). Meta-analyses were performed in R (version 4.5.0) using the meta-package using a random effect model with the DerSimonian-Laird estimator.^19^ For other outcomes, where heterogeneity in outcomes or study design did not allow for meta-analysis, a narrative approach was taken (i.e. qualitative synthesis of data).

### Certainty assessment

The quality of evidence was graded using the ‘Grades of Recommendation, Assessment, Development and Evaluation Working Group system’ (GRADE system).^20^ Evidence was graded as high, moderate, low, or very low quality.

## Results

### Selection process

**Figure 1:**
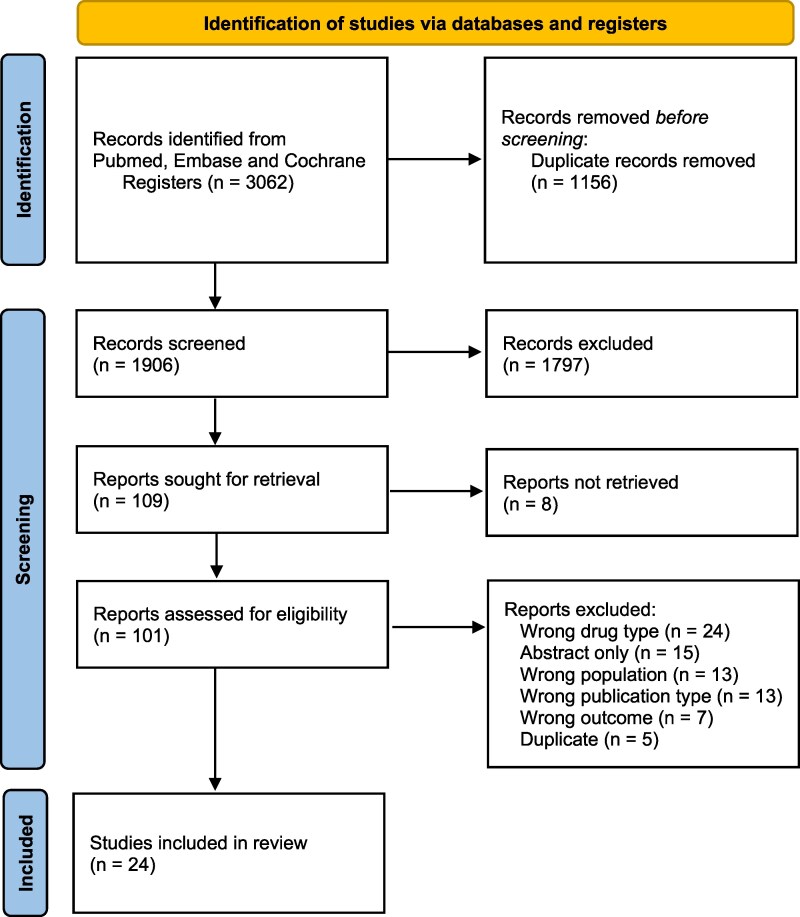
Flowchart of included studies.

The database search was conducted on 1 March 2025 and yielded a total of 3147 potentially eligible papers. After deduplication, screening was performed on 1906 abstracts and a subsequent 109 papers were selected for full text review (*Figure 1*).^21^ Out of the 109 papers selected for full-text review, the full-text manuscripts of 8 papers were not available. The remaining 101 manuscripts were retrieved and assessed for eligibility based on the predefined inclusion and exclusion criteria. In total, 24 studies were included for the present review. Excluded studies and the reason for exclusion are reported in the supplementary files (see Supplementary material online, *Table S5*). Homogeneity in study design and outcomes allowed a meta-analysis of five independent studies on mean heart rate during 24-h Holter monitoring and eight studies on maximum heart rate during exercise testing.

### Study characteristics

The 24 articles included consisted of 21 original sources of data and 4 articles reporting separate outcomes from the same clinical trial^3,22–24^ (*Table 2*). Of the 21 original studies, 6 studies were randomized crossover trials, 3 of which were open label,^9,11,15^ 2 were investigator blind^3,25^ and 1 was double blind.^15^ A further two studies were non-randomized open label crossover trials^8,13^ and one study was a multicentre randomized controlled (open label) trial with partial crossover.^12^ The remaining 12 studies were cohort studies or retrospective analyses.^26–36^  *Table 3* provides an overview of study characteristics and reported outcome measures. Supplementary material online, *Table S6* provides additional information on patient characteristics in the included studies.

**Table 3:** Included studies and characteristics

Study (first author, year)	Study type	Calcium channel blockers	Beta blockers	Follow up duration	Outcome measure
Wang *et al*. (1980)^8^	Non-randomized open label crossover trial	Verapamil 3dd 40 mg	Pindolol 5 mg	1 week	Ventricular heart rate (maximum, minimum, average)
Myers *et al*. (1987)^13^	Non-randomized open label crossover trial	Diltiazem 6dd 60 mg (4dd 30 mg for one patient)	Celiprolol 600 mg	1 week	Ventricular heart rate(maximum, minimum, average), perceived exertion, gas exchange
James *et al*. (1989)^25^	Randomized investigator blind crossover trial	Verapamil 40 mg (+digoxin)	Pindolol 5–15 mg (+digoxin)	1 week	Ventricular heart rate (maximum, minimum, average), symptoms (palpitations, breathlessness)
Matsuda *et al*. (1991)^14^	Randomized open label crossover trial	Verapamil 3dd 80 mg	Propranolol 3dd 20 mg	1 week	Ventricular heart rate during exercise (rest, anaerobic threshold, peak exercise), oxygen uptake during exercise
Dahlstrom *et al*. (1992)^15^	Randomized double-blind crossover trial	Diltiazem 60 mg	Propranolol 20 mg	4 weeks	Ventricular heart rate (during rest and exercise), maximum workload during exercise, exercise time, subjective sensation of wellbeing
Koh *et al*. (1995)^9,10^	Randomized open label crossover trial	Diltiazem 2dd 90 mg (+digoxin 0.125–0.5 mg)	betaxolol 1dd 20 mg (+digoxin 0.125–0.5 mg)	4 weeks	Maximum exercise tolerance, Ventricular heart rate (during rest and exercise) (24 h ECG in 15 patients)
Farshi *et al*. (1999)^11^	Randomized open label crossover trial	Diltiazem 1dd 240 mg (and diltiazem 240 mg + digoxin 0.25 mg)	atenolol 1dd 50 mg (and atenolol 50 mg + digoxin 0.25 mg)	2 weeks	Ventricular heart rate (24 h mean, max, during exercise, daytime, night-time), exercise duration.
Olshansky *et al*. (2004)^26^	Observational retrospective analysis derived from a multicentre randomized controlled open label trial	Diltiazem or verapamil	All beta blockers besides sotalol	3.5 (± 1.3) years	Rate control at rest (<80 b.p.m.), rate control during exertion (<110 b.p.m. 6 min walk test), overall rate control (rest and exertion), adverse drug effects.
Tsuneda *et al*. (2006)^12^	Multicentre randomized controlled (open label) trial with partial crossover	Verapamil (mean dosage: 120 mg) (dosages titrated to achieve heart rate 60–80 b.p.m.)	Bisoprolol (mean dosage 3.75 mg), atenolol (mean dosage 30 mg) or metoprolol (mean dosage 40 mg) (dosages titrated to achieve heart rate 60–80 b.p.m.)	≥ 1 month	Ventricular heart rate (maximum, minimum, average), exercise duration, max HR during exercise, quality of life SF 36
Climent *et al*. (2010)^27^	Prospective observational trial	Verapamil	Beta blockers	1 day	Mean heart rate (baseline and after drug admission)
Ulimoen *et al*. (2013)^3^	Randomized, investigator-blind, crossover trial	Diltiazem 360 mg, verapamil 240 md	Metoprolol 100 mg, carvedilol 25 mg	3 weeks	Ventricular heart rate (at rest, 24 h mean, daytime, night-time), symptom frequency and severity
Scheuermeyer *et al*. (2013)^28^	Retrospective cohort study	Diltiazem or verapamil (all dosages)	All beta blockers besides sotalol (all dosages)	30 days after discharge	Hospital admission, length of stay, heart rate of admitted/discharged patients (*Table 6* secondary outcomes)
Ulimoen *et al*. (2014)^22,37^	Randomized, investigator-blind, crossover trial	Diltiazem 360 mg, verapamil 240 md	Metoprolol 100 mg, carvedilol 25 mg	3 weeks	Peak oxygen uptake during exercise, NT-proBNP (at rets, during exercise), heart rate (during exercise, peak heart rate)
Corino *et al*. (2015, Feb)^23^	Randomized, investigator-blind, crossover trial	Diltiazem 360 mg, verapamil 240 md	Metoprolol 100 mg, carvedilol 25 mg	3 weeks	Mean heart rate during peak rate reducing effect (20 min at 2 p.m.)
Horjen *et al*. (2016)^24^	Randomized, investigator-blind, crossover trial	Diltiazem 360 mg, verapamil 240 md	Metoprolol 100 mg, carvedilol 25 mg	3 weeks	High sensitive troponin I (at rest, during exercise)
Yu *et al*. (2018)^29^	Observational cohort study	Verapamil or diltiazem	Acebutolol, arotinolol, atenolol, bevantolol, bisoprolol, carvedilol, celiprolol, labetalol, metoprolol, nadolol, nebivolol, and propranolol.	4.5 (± 1.2) years	All cause death
You *et al*. (2018)^30^	Observational cohort study	Verapamil or diltiazem	Selective: atenolol, bisoprolol, nebivolol, and metoprolol. Non-selective BBs included propranolol, bevantolol, carvedilol, and betaxolol	4.2 (± 3.2) years	All cause death
Zaman *et al*. (2021)^31^	Observational retrospective analysis derived from a multicentre randomized controlled open label trial	Diltiazem or verapamil	All beta blockers besides sotalol	Not specified for subgroup, max 5 years	Time to first hospitalization and time to death from any cause, torsade de pointes, VT, cardiac arrest, ischaemic stroke, major bleed
Koldenhof *et al*. (2022)^32^	Observational retrospective analysis derived from a multicentre randomized controlled open label trial	Verapamil	All beta blockers besides sotalol	3.1 years	Time to first electrical or chemical cardioversion or AF ablation whichever came first. Secondary: death, arrhythmic events, Heart failure, ACS, ischaemic thromboembolic event, life-threatening effects of drugs
Sherf *et al*. (2022)^33^	Observational cohort study	Diltiazem or verapamil	All beta blockers besides sotalol	Until discharge	Hospitalization length, time to heart rate <100 b.p.m.,
Barcia *et al*. (2024)^34^	Observational cohort study	Diltiazem or verapamil	All beta blockers besides sotalol	3.3 years	Hospitalizations due to poor heart rate control (subgroups based on eGFR)
Koldenhof *et al*. (2023)^36^	Observational retrospective analysis derived from a multicentre randomized controlled open label trial	Diltiazem or verapamil	All beta blockers besides sotalol	119 days	Bradycardia during sinus rhythm, lenient rate control during AF
Menichelli *et al*. (2024)^35^	Observational cohort study	Diltiazem or verapamil	All beta blockers besides sotalol	31 months	Cardiac hospitalisations
Enge *et al*. (2024)^16^	Parallel-group, randomized, investigator-blinded clinical trial	Diltiazem 360 mg	Metoprolol 100 mg	6 months	NT-proBNP, heart rate (rest and 24 h) peak VO_2_

### Assessment of risk of bias

Risk of bias assessment (*Table 4*) showed some risk of bias in the majority of randomized studies. Patient dropout partway through the crossover design resulted in some missing outcome data, potentially skewing outcome data, leading to ‘some’ risk of bias in the ‘Missing outcome data’ domain. Most non-randomized studies had a serious risk of bias (*Table 5*), with one study having critical risk of bias.

**Table 4:** Risk of bias assessment randomized trials using risk of bias tool 2

	Domain 1	Domain 2	Domain 3	Domain 4	Domain 5	Overall
James *et al*. (1989)^25^	Low	Some	Some	Low	Some	Some
Matsuda *et al*. (1991)^14^	Some	Some	High	Low	Some	High
Dahlstrom *et al*. (1992)^15^	Some	Low	Low	Low	Some	Some
Koh *et al*. (1995)^9,10^	Some	Low	Some	Low	Some	Some
Farshi *et al*. (1999)^11^	Low	Low	Low	Low	Some	Some
Tsuneda *et al*. (2006)^12^	Some	Low	Low	Some	Some	Some
Ulimoen *et al*. (2013)^3^	Low	Low	Some	Low	Low	Some
Ulimoen *et al*. (2014)^22,37^	Low	Low	Some	Low	Low	Some
Corino *et al*. (2015, feb)^23^	Low	Low	Some	Low	Low	Some
Horjen *et al*. (2016)^24^	Low	Low	Some	Low	Low	Some
Enge *et al*. (2024)^16^	Low	Low	Low	Low	Low	low

Domains:

1. Bias arising from the randomization process.

2. Bias due to deviations from intended interventions.

3. Bias due to missing outcome data.

4. Bias in measurement of the outcome.

5. Bias in selection of the reported result.

**Table 5:** Risk of bias assessment non-randomized trials using ROBINS-I

	Domain 1	Domain 2	Domain 3	Domain 4	Domain 5	Domain 6	Domain 7	Overall
Wang *et al*. (1980)^8^	Low	Serious	Low	Low	Low	Low	Low	Serious
Myers *et al*. (1987)^13^	Low	Low	Low	Low	Low	Moderate	Low	Moderate
Olshansky *et al*. (2004)^26^	Serious	Serious	Low	Low	Moderate	Low	Low	Serious
Climent *et al*. (2010)^27^	Serious	NI	Low	Low	Low	Low	Low	Serious
Scheuermeyer *et al*. (2013)^28^	Serious	Serious	Low	Low	Low	Low	Low	Serious
You *et al*. (2018)^30^	Serious	Serious	Low	Low	NI	Low	Moderate	Serious
Yu *et al*. (2018)^29^	Serious	Serious	Moderate	Low	NI	Low	Moderate	Serious
Zaman *et al*. (2021)^31^	Moderate	Serious	Low	Low	Moderate	Low	Low	Serious
Koldenhof *et al*. (2022)^32^	Serious	Serious	Low	Low	Low	Low	Low	Serious
Sherf *et al*. (2022)^33^	Serious	Serious	Low	Low	NI	Moderate	Moderate	Serious
Barcia *et al*. (2024)^34^	Serious	Critical	Low	Low	Serious	Serious	Moderate	Critical
Koldenhof *et al*. (2023)^36^	Serious	Moderate	Low	Low	Moderate	Low	Moderate	Serious
Menichelli *et al*. (2024)^35^	Serious	Serious	Serious	Low	NI	Moderate	Moderate	Serious

Risk Of Bias In Non-Randomized Studies—of Interventions (ROBINS-I).

Domains:

1. Bias due to confounding.

2. Bias in selection of participants into the study.

3. Bias in classification of interventions.

4. Bias due to deviations from intended interventions.

5. Bias due to missing data.

6. Bias in measurement of outcomes.

7. Bias in selection of the reported result.

### Quantitative synthesis

**Figure 2:**
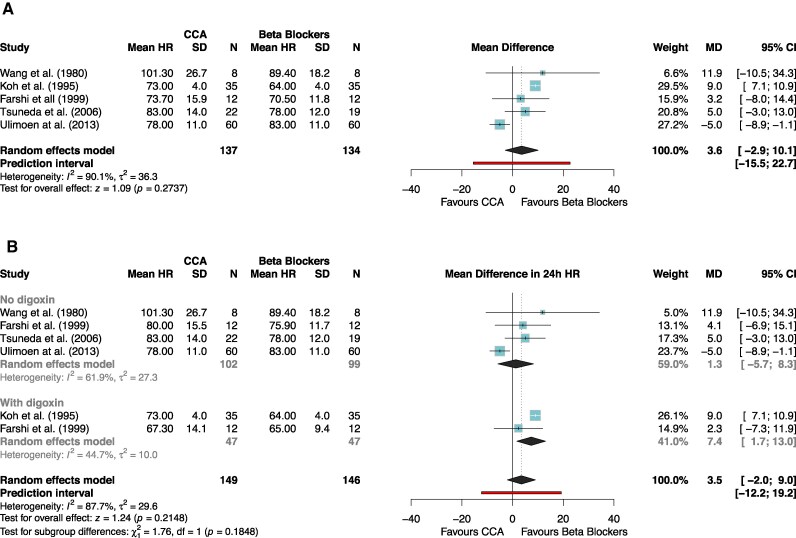
(*A*) meta-analysis of 24 h heart rate. Forest plots of the mean heart rate during 24 h Holter monitoring. *Lower heart rate was defined as being favourable. (*B*) meta-analysis of 24 h heart rate, with and without digoxin. Forest plots of the mean heart rate during 24 h Holter monitoring with subgroups based on the addition of digoxin. *Lower heart rate was defined as being favourable.

Of five studies included in the mean 24 h heart rate meta-analysis, three studies compared calcium channel blockers with beta blockers without the addition of digoxin, one study with the addition of digoxin, and one study had a crossover design comparing calcium channel blockers and beta blockers both with and without digoxin. Clinical diversity was low (see Supplementary material online, *Table S6*) and all five studies were included in the main analysis (*Figure 2A*) with a sub-analysis of studies with and without digoxin (*Figure 2B*). Moderate quality of evidence revealed that mean heart rate during 24 h Holter monitoring was not significantly different between calcium channel blockers vs. beta blockers (MD 3.6 b.p.m., 95% CI: −2.9 to 10.1, 95% PI: −15.5 to 22.7, *P* = 0.27, *Table 6*). Subgroup analysis with and without digoxin revealed no significant differences (*P* = 0.18, *Figure 2B*). Sensitivity analysis using the restricted maximum likelihood model revealed no significant differences to the main results (see Supplementary material online, *Figure S1*).

**Table 6:** Summary of findings of the randomized controlled trials with quality of evidence assessment

Outcome	Subjects, *N* (studies)	SMD (95% CI)	Quality of evidence (GRADE)
24 h mean heart rate	137 (5)	3.6 (−2.9; 10.1)	Moderate^a^
Maximum heart rate during exercise	210 (8)	10.5 (3.3; 17.7)	High

SMD, standardized mean difference; GRADE, Grading of Recommendations, Assessment, Development and Evaluation; CCA, calcium channel antagonist; BB, beta blocker.

^a^Downgraded one level for inconsistency: substantial methodological or clinical heterogeneity that could not be accounted for in analyses.

**Figure 3:**
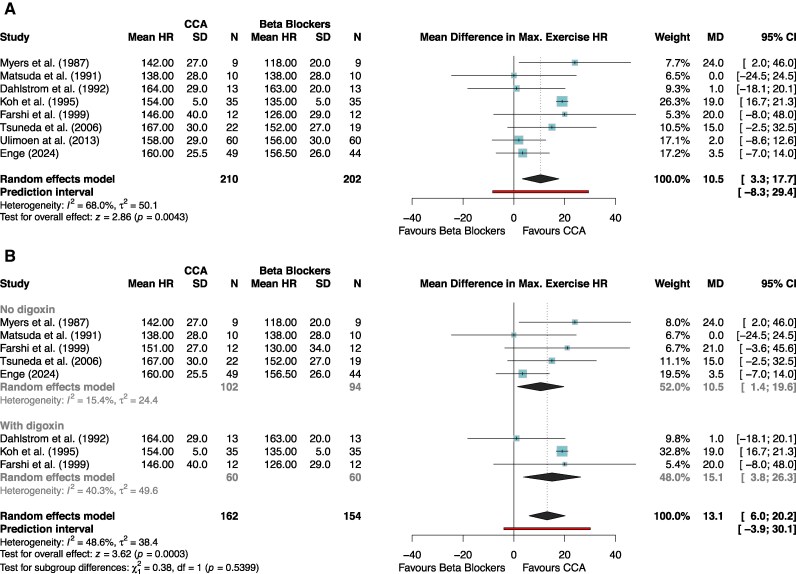
(*A*) meta-analysis of max heart rate during exercise. Forest plots of the maxim heart rate during exercise. *Higher heart rate was defined as being favourable. (*B*) meta-analysis of max heart rate during exercise, with and without digoxin. Forest plots of the maxim heart rate during exercise dub subgroups based on the addition of digoxin. *Higher heart rate was defined as being favourable.

Of the eight studies included in the maximum heart rate during exercise, four studies compared calcium channel blockers with beta blockers without the addition of digoxin, three studies with the addition of digoxin, and one study had a crossover design comparing calcium channel blockers and beta blockers both with and without digoxin Clinical diversity was low (see Supplementary material online, *Table S6*). High quality of evidence revealed maximum heart rate during exercise to be significantly higher in patients taking calcium channel blockers vs. beta blockers (MD 10.5 b.p.m., 95% CI: 3.3 to 17.7, 95% PI: −8.3 to 29.4, *P* < 0.01, *Figure 3A*, *Table 6*). Subgroup analysis with and without digoxin revealed no significant differences (*P* = 0.54, *Figure 2B*).

### Qualitative synthesis

#### Heart rate and rate control

In addition to the studies included in the meta-analysis for 24 h heart rate, four studies reported rate control related outcomes. One of these studies was randomized and had ‘some’ risk of bias,^25^ while the remaining three were non-randomized and had a high risk of bias.^26,27,36^ James *et al*.^25^ reported that the combination of beta blocker and digoxin was significantly more effective at reducing maximum heart rate compared with the combination of calcium channel blocker and digoxin as measured during 24 h heart rate monitoring (*P* < 0.02). However, the study by James *et al*. presented heart rate data only as a visual graph without providing numerical values, and therefore could not be included in the meta-analysis. Olshansky *et al*.^26^ retrospectively analysed achieving ‘strict’ rate control (i.e. heart rate <80 b.p.m. in rest and <110 b.p.m. during mild exercise) in patients treated with calcium channel blockers and beta blockers with and without digoxin. Strict rate control was achieved in 70% of patients starting rate control with beta blockers and in 54% of patients with calcium channel blockers both with and without digoxin (no *P* value reported). Koldenhof *et al*.^36^ performed a retrospective analysis in the same database including patients with non-permanent AF and reported no difference in achieving lenient rate control during AF (92% vs. 92%, *P* = 1.00), but there were fewer episodes of bradycardia during sinus rhythm for patients using calcium channel blockers (17% vs. 32%, *P* < 0.01). Climent *et al*.^27^ calculated mean RR intervals during 24 h Holter monitoring of a selection of three patients with calcium channels blockers and three patients with beta blockers but performed no statistical tests.

#### Exercise tolerance and maximum oxygen uptake

Multiple studies reported exercise-related outcomes other than maximum heart rate. However, heterogeneity in study design and reported outcomes did not allow for a meta-analysis. Four studies reported peak volume of oxygen (VO_2_) max during exercise, using different exercise protocols and duration of drug usage before the exercise test.^13,14,16,37^ Two studies reported on measured exercise capacity in watts or metabolic equivalents^9,15^ and one study reported perceived exertion during exercise.^13^

Myers *et al*.^13^ a study with a ‘moderate’ risk of bias, reported a significant reduction In maximum oxygen uptake (VO_2_ max) during peak exercise of 3 mL of oxygen per kilogram of body weight per minute (mL/kg/min) in patients receiving beta blockers compared with calcium channel blockers (*P* < 0.01). Similarly, Ulimoen *et al*.^37^ a study with ‘some’ risk of bias, reported a significant difference in peak VO_2_ of 2.85 mL/kg/min in favour of calcium channel blockers over beta blockers (*P* < 0.001). Enge *et al*. with a ‘low’ risk of bias, reported that patients with calcium channel blockers had higher peak VO_2_ at 1 month follow-up compared with patients with beta blockers (0.92 mL/kg/min, *P* = 0.034), but there were no differences at 6 months (0.69 mL/kg/min, *P* = 0.126). Lastly, Matsuda *et al*.^14^ reported no difference in VO_2_ max between patients using beta blockers and calcium channel blockers (no *P* value reported and a ‘serious’ risk of bias). Dahlström *et al*. and Koh *et al*. both with ‘some’ risk of bias, reported no difference in maximum workload in watts and metabolic equivalents respectively during exercise test between patients using beta blockers and calcium channel blockers (Dahltröm *P* = 0.89, Koh *P* > 0.05).^10,15^ Additionally to VO_2_ max, Meyers *et al*.^13^ reported increased perceived exertion during 80% of maximum exercise for patients taking beta blockers compared with calcium channel blockers (difference in rate of perceived exertion = 2, *P* < 0.01).

#### Symptoms

Four studies reported on quality of life or symptoms, all of them being randomized studies, 1 with ‘low’ and 3 with ‘some’ risk of bias.^12,16,22,25^ Ulimoen *et al*. reported a reduction of symptom severity and frequency in patients using diltiazem compared with baseline (*P* < 0.001) and a reduction in symptom frequency in patients using verapamil compared with baseline (*P* = 0.012). There were no differences in symptom severity or frequency between patients using beta blockers compared with baseline.^3^ Similarly, Enge *et al*. reported a reduction in symptom severity and frequency in patients using diltiazem at both 1 month and 6 months follow-up compared with baseline (all <0.005). In patients receiving beta blockers there was no significant reduction in symptom severity or frequency compared with baseline.^16^ One study reported that patients with calcium channel blockers experienced less breathlessness but there were no differences in palpitations compared with patients using beta blockers.^25^ Tsuneda *et al*.^12^ reported that in patients with calcium channel blockers, quality of life as measured by Short Form-36 and Atrial Fibrillation Quality of Life Questionnaire significantly improved compared with baseline (physical functioning, role function-emotional, mental component summary all *P* < 0.05, role function-physical and variety and frequency of symptoms <0.01), while patients with beta blockers showed no improvement in quality of life.

#### Atrial fibrillation progression

One non-randomized study with a ‘serious’ risk of bias, reported one difference in progression of AF between calcium channel blockers and beta blockers. Koldenhof *et al*.^32^ reported that patients using calcium channel blockers had less AF progression, defined as the need for electrical or chemical cardioversion or AF ablation, compared with patients using beta blockers (17% vs. 33%, *P* = 0.038).

#### Hospital admissions

Four non-randomized studies with a ‘serious’ risk of bias reported on need for hospital admission or length of hospitalization.^31,33–35^ In the study of Zaman *et al*.^31^ there were no differences in hospital admissions between calcium channel blockers and beta blockers. Menichelli *et al*.^35^ detected no association between calcium channel blockers or beta blockers an risk of hospitalization. In patients with an estimated glomerular filtration rate (eGFR) < 30, calcium channel blockers were associated with an increased risk of hospitalizations due to poor heart rate control compared with patients using beta blockers in a retrospective analysis by Barcia *et al*.^34^ (HR 4.53, 95% CI 1.19–17.18; *P* = 0.026). The same effect was not found in patients with an eGFR between 30 and 60 or in patients with an eGFR >60. Sherf *et al*.^33^ reported no difference in duration of hospitalization or spontaneous cardioversion during hospitalization between beta blockers and calcium channel blockers.

#### Death

Three non-randomized studies with a ‘serious’ risk of bias, analysed the risk of all-cause mortality in patients with AF.^29–31^ In Korean insurance data, You *et al*.^30^ reported a reduction in all-cause mortality in patients with AF and obstructive lung disease treated with non-selective and selective beta blockers compared with calcium channel blockers (HR 0.84, 95% CI 0.75–0.94 and HR 0.85, 95% CI 0.77–0.95, respectively). Using the same data, Yu *et al*.^29^ observed that beta blockers were associated with a reduced risk of death in patients with AF and with heart failure (adjusted HR 0.63, 95% CI 0.50–0.79), but the same effect was not found in patients using calcium channel blockers. Zaman *et al*.^31^ showed no difference in death after adjusting for baseline differences when comparing calcium channel blockers and beta blockers.

#### Adverse drug effects

Two non-randomized studies with a ‘serious’ risk of bias analysed adverse effects between calcium channel blockers and beta blockers.^26,31^ Olshansky reported adverse effects of drugs but performed no statistical testing. Overall both drugs were reported as well tolerated.^26^ Zaman, analysing the same data as Olshansky, reported no significant differences in adverse events.

#### Sensitivity analysis

Sensitivity analyses of both mean 24 h heart rate and maximum heart rate during exercise are displayed in the supplementary files (see Supplementary material online, *Tables S7* and *S8*, respectively). Results of the 24 h mean heart rates changes significantly when omitting Ulimoen *et al*. from the meta-analysis resulting in a significantly higher mean 24 h heart rate in patients using calcium channel blockers (MD 8.4 b.p.m., 95% CI 0.0 to 8.5, *P* < 0.001) and a substantial decrease in statistical heterogeneity (*I*^2^ from 90% to 0%). Excluding others studies did not substantially alter the results. Sensitivity analysis of heart rate during exercise did not reveal any differences compared with the main results.

## Discussion

This meta-analysis shows that beta blockers and calcium channel blockers are equally effective at reducing mean heart rate in patients with persistent or permanent AF. These results were consistent with and without the addition of digoxin. During exercise, mean maximum heart rate was significantly higher in patients receiving calcium channel blockers compared with patients receiving beta blockers. Some of the randomized studies showed simultaneous increase in VO_2_ max during exercise in patients receiving calcium channel blockers.

## Rate control

This meta-analysis demonstrates that both calcium channel blockers and beta blockers can be effectively used to control heart rate in patients with AF. Previous meta-analyses have demonstrated that calcium channel blockers and beta blockers can both be used for acute rate control.^38–40^ To the best of our knowledge, this is the first meta-analysis comparing calcium channel blockers and beta blockers for rate control in a non-acute setting.

Overall, most trials included in the meta-analysis had comparable study designs, with the majority using a crossover design that incorporated a washout period between initiation of rate control drugs and Holter monitoring and exercise testing. Between studies, there was considerable variation in the types of beta blockers used. Over time, both clinical practice and research have increasingly favoured newer-generation, selective beta blockers due to their improved side-effect profiles.^41^ However, earlier studies have shown that non-selective beta blockers are not inferior in reducing heart rate compared with selective beta blockers.^42^ Therefore, it is unlikely that the change in beta blocker type substantially influenced the overall findings on rate heart rate.

## Qualitative synthesis

Several outcomes could not be included in the meta-analysis and were instead reported narratively. Heterogeneity in study design, population and measured outcomes in these outcomes were too great and often the number of studies reporting them were insufficient to allow for meta-analysis. In addition, almost all non-randomized studies had a ‘serious’ risk of bias and should be interpreted with caution. The results of the qualitative synthesis should therefore be seen as hypothesis generating.

Rate control during exercise: Maximum heart rate during exercise testing was significantly higher in patients using calcium channel blocker compared with those using beta blockers. Interestingly, three studies reported increased peak oxygen uptake during exercise in patients receiving calcium channel blockers compared with patients receiving beta blockers. Earlier studies have shown that impaired chronotropic response reserve, the change in heart rate from rest to peak exercise, is a major contributor to reduced peak oxygen uptake.^43,44^ This is especially relevant in patients with heart failure with preserved ejection fraction, in whom cardiac output cannot be significantly increased by increasing stroke volume.^43,45^ Indeed peak oxygen uptake was directly correlated to peak heart rate during exercise in one of the studies included in the meta-analysis.^22^ The difference in peak oxygen uptake between patients using calcium channel blockers and those using beta blockers may, therefore, be explained by the ability of calcium channel blockers to allow a higher heart rate during exercise. In this systematic review no meta-analysis could be performed on peak oxygen uptake during exercise, and it remains uncertain whether this observed difference translates into any clinical benefit in clinical practice.

Quality of life: three randomized studies reported that calcium channel blockers reduce AF symptom severity and frequency, or improved quality of life as measured by the SF-36 score. In contrast two non-randomized studies with a high risk of bias reported no difference in adverse effects between groups. These results suggest a potentially meaningful difference between calcium channel blockers and beta blockers in suppressing AF related symptoms. Since no meta-analysis could be performed, this systematic review does not provide decisive evidence favouring one rate control drug over the other. But, in cases where patients remain symptomatic on one rate control agent, switching to the alternative drug may be considered.

AF progression: one non-randomized study with a high risk of bias reported that patients with calcium channel blockers had less AF progression compared with patients treated with beta blockers. These results have not yet been replicated, and their clinical relevance remains uncertain. A randomized trial would have to be performed comparing the effect of calcium channel blockers and beta blockers on AF progression.

Hospital admissions and all-cause death: four non-randomized studies reported on hospital admissions or length of hospitalization and report conflicting results. All studies reporting on hospital admissions had a serious risk of bias and offered no convincing benefit of one drug over the other. Similarly, two non-randomized studies with a serious risk of bias report on all cause death. One reporting an association between beta blockers reduced risk of all cause death and one study reporting no difference between calcium channel blockers and beta blockers. No high level of evidence was found proving a significant reduction in mortality between groups. In our opinion, the available data fails to provide any evidence in favour of either calcium channel blockers or beta blockers for reducing hospital admissions and mortality in patients with AF and should not be used to inform therapeutic recommendations.

## Heterogeneity

Although no significant differences between the subgroups with and without digoxin were observed, the overall heterogeneity could mainly be explained by the subgroup analysis. Inclusion and exclusion criteria were relatively similar between studies, but the type and dosages of calcium channel blockers and beta blockers varied. Exclusion of Ulimoen *et al*. significantly altered the results of the 24-h mean heart rate analysis, reducing the *I*^2^ from 90% to 0%. A possible explanation could be differences in the dosages of calcium channel blockers and beta blockers used across the trials. Calcium channel blocker dosages ranged from as low as 40 mg of verapamil (maximum recommended dose: 480 mg daily) to 360 mg of diltiazem (maximum recommended dose: 540 mg daily).

With 60 patients in each group, Ulimoen *et al*. represents the largest of the trials and therefore carries considerable weight in the 24-h heart rate meta-analysis. Coincidentally, the drug dosages used in their trial were at the high end of the dosing spectrum. In the calcium channel blocker group, patients were treated with 360 mg of diltiazem or 240 mg of verapamil, representing ∼67% and 50% of the maximum recommended doses, respectively. These were compared with patients receiving 100 mg of metoprolol or 25 mg of carvedilol, both representing about 50% of their maximum recommended doses. In the second largest trial conducted by Koh *et al*. with 35 patients in each group, the calcium channel blocker group was treated with 180 mg of diltiazem daily, ∼33% of its maximum recommended dose and compared with betaxolol 20 mg daily, which represents 100% of its maximum recommended dose. It is possible that in many of the earlier trials, sub-therapeutic doses of calcium channel blockers may have contributed to a perceived superiority of beta blockers after omitting Ulimoen *et al*. The sensitivity analysis of the maximum heart rate during exercise meta-analysis, also including the trial by Ulimoen *et al*. showed no significant differences in outcome of heterogeneity from the main results.

## Strengths and limitations

This is the first meta-analysis showing effects of calcium channel blockers and beta blockers on mean heart rate and maximum heart rate during exercise. The quality of evidence was moderate for the 24 h heart rate meta-analysis. There was significant heterogeneity between the included studies, resulting in a downgrade of the quality of evidence. For maximum heart rate during exercise the quality of evidence was high. In addition to a meta-analysis, a qualitative synthesis was performed for other less reported outcomes thereby further exploring differences in calcium channel blockers and beta blockers. Several important limitations should be mentioned. Firstly, there was substantial heterogeneity between studies included in the meta-analyses. Studies mainly differed in types of beta blockers used and differences in dosages used for both beta blockers and calcium channel blockers. Sensitivity analysis did reveal significant differences in outcome after excluding Ulimoen *et al*., the largest and most recent study. Furthermore, almost all non-randomized studies had at least a serious risk of bias. Consistent information on cardiovascular outcomes such hospitalization, heart failure progression, and mortality were lacking. Finally, due to the limited number of studies, funnel plots were not created, and an assessment of reporting bias was not performed. Considering the limitations of this analysis, further randomized controlled trials comparing channel blockers and beta blockers for rate control are needed.

## Conclusion

In this meta-analysis of 10 randomized controlled studies, calcium channel blockers and beta blockers showed no difference in reducing mean heart rate during 24 h heart rate monitoring in patients with persistent and permanent AF. Maximum heart rate during exercise testing was significantly higher in patients taking calcium channel blockers compared with those taking beta blockers. Several studies reported higher peak oxygen uptake in patients with calcium channel blockers compared with patients with beta blockers however, these results should be considered hypothesis-generating. Due to a lack of homogeneous studies, meta-analysis of peak oxygen uptake, symptoms, hospital admissions, adverse events, death and AF progression could not be performed and were, thus, limited to qualitative synthesis.

## Supplementary Material

oeag062_Supplementary_Data

## Data Availability

Data will be shared on reasonable request.
