# Supplementary material for: Rate control treatment with calcium channel blockers and beta blockers for patients with atrial fibrillation: a systematic review and meta-analysis
Source: Eur Heart J Open. 2026 Apr 16;6(2):oeag062. doi: 10.1093/ehjopen/oeag062 (PMC13155099; doi:10.1093/ehjopen/oeag062)
Supplement: oeag062_Supplementary_Data [file oeag062_supplementary_data.docx]

**Supplementary files**

**Rate control treatment with calcium channel blockers and beta blockers for patients with atrial fibrillation: a systematic review and meta-analysis.**

Tim Koldenhof^1, 2^, Barzi Gareb^3^, Marcelle D. smit^1^, Thijmen SA. Bergwerff^2^, Isabelle C. van Gelder^2^, Michiel Rienstra*^2^, Robert G. Tieleman*^1,2^

Supplementary 1 - Search strategy

Table S1: **Pubmed**Date run: march 1st 2025

| ID | Search | Hits |
| --- | --- | --- |
| #1 | ("Atrial Fibrillation"[Mesh] OR "Atrial Flutter"[Mesh] OR ((atrial[tiab] OR atrium[tiab] OR auricular[tiab]) AND (fibrillation*[tiab] OR fibrilation*[tiab] OR flutter*[tiab] OR arrythmia*[tiab])) OR AF[tiab]) | 144.187 |
| #2 | ("Calcium Channel Blockers"[Mesh] OR “calcium channel block*”[tiab] OR CCB[tiab] OR CCBs[tiab] OR “calcium channel antagonist*”[tiab] OR ((calcium[tiab]) AND (antagonist*[tiab] OR inhibitor*[tiab] OR block*[tiab])) OR “calcium entry block*”[tiab] OR "Calcium Channel Blockers" [Pharmacological Action] OR verapamil[tiab] OR diltiazem [tiab]) | 197.296 |
| #3 | ("Adrenergic beta-Antagonists"[Mesh] OR “adrenergic beta antagonist*”[tiab] OR “beta adrenergic receptor blocking agent*”[tiab] OR "Adrenergic beta-Antagonists" [Pharmacological Action] OR ((beta[tiab]) AND (blocker*[tiab] OR blockader*[tiab] OR antagonist*[tiab])) OR "Metoprolol"[Mesh] OR metoprolol*[tiab] OR "Bisoprolol"[Mesh] OR bisoprolol*[tiab] OR "Atenolol"[Mesh] OR atenolol*[tiab] OR "Acebutolol"[Mesh] OR acebutolol*[tiab] OR "Carvedilol"[Mesh] OR carvedilol*[tiab] OR "esmolol" [Supplementary Concept] OR esmolol*[tiab] OR "Labetalol"[Mesh] OR labetalol*[tiab] OR "Propranolol"[Mesh] OR propranolol*[tiab] OR "Nebivolol"[Mesh] OR nebivolol[tiab]) | 156.017 |
| #4 | #1 AND #2 AND #3 | 980 |

Table S2: Embase

Date run: march 1st 2025

| ID | Search | Hits |
| --- | --- | --- |
| #1 | ('atrial fibrillation'/exp/mj OR 'atrial fibrillation':ab,ti OR 'atrial flutter'/exp/mj OR 'atrial flutter' OR 'atrial flutter':ab,ti OR ((atrial:ab,ti OR atrium:ab,ti OR auricular:ab,ti) AND (fibrillation*:ab,ti OR fibrilation*:ab,ti OR flutter*:ab,ti OR arrythmia*:ab,ti)) OR af:ab,ti) NOT 'conference abstract'/it | 147.748 |
| #2 | ('calcium channel blockers'/exp/mj OR 'calcium channel block*':ab,ti OR ccb:ab,ti OR ccbs:ab,ti OR 'calcium channel antagonist*':ab,ti OR (calcium:ab,ti AND (antagonist*:ab,ti OR inhibitor*:ab,ti OR block*:ab,ti)) OR 'calcium entry block*':ab,ti OR 'verapamil'/exp/mj OR 'verapamil':ab,ti OR 'diltiazem'/exp/mj OR 'diltiazem':ab,ti) NOT 'conference abstract'/it | 212.215 |
| #3 | ('beta adrenergic receptor blocking agent'/exp/mj OR 'beta adrenergic receptor blocking agent*':ti,ab OR 'adrenergic beta antagonist*':ab,ti OR ((beta:ab,ti OR β:ti,ab) AND (blocker*:ab,ti OR blockader*:ab,ti OR antagonist*:ab,ti)) OR 'metoprolol'/exp/mj OR metoprolol*:ab,ti OR 'bisoprolol'/exp/mj OR bisoprolol*:ab,ti OR 'atenolol'/exp/mj OR atenolol*:ab,ti OR 'acebutolol'/exp/mj OR acebutolol*:ab,ti OR 'carvedilol'/exp/mj OR carvedilol*:ab,ti OR 'esmolol'/exp/mj OR esmolol*:ab,ti OR 'labetalol'/exp/mj OR labetalol*:ab,ti OR 'propranolol'/exp/mj OR propranolol*:ab,ti OR 'nebivolol'/exp/mj OR nebivolol:ab,ti) NOT 'conference abstract'/it | 197.315 |
| #4 | #1 AND #2 AND #3 | 1355 |

Table S3: Web of science

Date run: march 1st 2025

| ID | Search | Hits |
| --- | --- | --- |
| #1 | TS=("atrial fibrillation" OR "Atrial Flutter" OR ((atrial OR atrium OR auricular) AND (fibrillation OR fibrilation OR flutter OR arrythmia))) | 145.104 |
| #2 | TS=("Calcium Channel Blockers" OR "Calcium Channel Block*" OR CCB OR CCBs OR "calcium channel antagonist*" OR ((calcium) AND (antagonist* OR inhibitor* OR block*)) OR “calcium entry block*” OR verapamil OR diltiazem) | 180.924 |
| #3 | TS=("Adrenergic beta-Antagonists" OR “adrenergic beta antagonist*"OR ((beta) AND (blocker* OR blockader* OR antagonist*)) OR Metoprolol OR Bisoprolol OR Atenolol OR Acebutolol OR Carvedilol OR esmolol OR Labetalol OR Propranolol OR Nebivolol) | 164.359 |
| #4 | #1 AND #2 AND #3 | 646 |

Table S4: Chochrane

Date run: march 1st 2025

| ID | Search | Hits |
| --- | --- | --- |
| #1 | ([mh "atrial fibrillation"] OR [mh "atrial flutter"] OR "atrial fibrillation":ti,ab,kw OR ((atrial OR atrium OR auricular) AND (fibrillation* OR fibrilation* OR flutter* OR arrythmia*)):ti,ab,kw) | 17.634 |
| #2 | ([mh "calcium channel blocker"] OR “calcium channel block*”:ti,ab,kw OR (CCB OR CCBs OR "calcium channel antagonist"):ti,ab,kw OR(("calcium")AND ("antagonist*" OR "inhibitor*" OR "block*")):ti,ab,kw OR ("calcium entry block" OR "verapamil" OR "diltiazem"):ti,ab,kw) | 10.470 |
| #3 | ([mh "Adrenergic beta-antagonist"] OR (("beta") AND ("blocker*" OR "blockader*" OR "antagonist*" )):ti,ab,kw OR ("metoprolol" OR "Bisoprolol" OR "Atenolol" OR "Acebutolol" OR "Carvedilol" OR "esmolol" OR "Labetalol" OR "Propranolol" OR "Nebivolol"):ti,ab,kw) | 20.625 |
| #4 | #1 AND #2 AND #3 | 166 |

Table S5: Excluded articles with reasons for exclusion after full-text screening

| **Author** | **Reason for exclusion** | **Reference** |
| --- | --- | --- |
| Ahuja (1989) | Wrong population | (1) |
| Atzema (2017) | Wrong drug type | (2) |
| Balser (1998) | Wrong population | (3) |
| Balser (1998) | Duplicate | (4) |
| Bedford (2022) | Wrong population | (5) |
| Bertaglia (2001) | Wrong publication type (abstract only) | (6) |
| Bertaglia (2003) | Wrong drug type | (7) |
| Bosch (2021) | Wrong population | (8) |
| Botto (1999) | Wrong publication type (editorial) | (9) |
| Carter (2017) | Wrong publication type (protocol) | (10) |
| Chen (2016) | Wrong publication type (abstract only) | (11) |
| Compagner (2022) | Wrong drug type | (12) |
| Corino (2015) | Wrong outcome | (13) |
| Cemircan (2005) | Wrong drug type | (14) |
| Demir (2021) | Wrong drug type | (15) |
| Demircan (2005) | Wrong drug type | (16) |
| Desai (2014) | Wrong outcome | (17) |
| Feeney (2018) | Wrong drug type | (18) |
| Frick (2024) | Wrong outcome | (19) |
| Friedman (1986) | Wrong publication type (abstract only) | (20) |
| Fromm (2015) | Wrong population | (21) |
| Gloaguen (2019) | Wrong publication type (abstract only) | (22) |
| Hargrove (2021) | Wrong drug type | (23) |
| Harvey (2017) | Wrong population | (24) |
| Hasbrouck (2022) | Wrong population | (25) |
| Hassan (2007) | Wrong population | (26) |
| Hilleman (2003) | Wrong population | (27) |
| Hines (2016) | Wrong outcome | (28) |
| Hirschy (2018) | Wrong publication type (abstract only) | (29) |
| Hirschy (2019) | Wrong population | (30) |
| Kanorskii (2002) | Wrong population type (editorial) | (31) |
| Kapustova (2023) | Wong drug type | (32) |
| Karaca (2007) | Wrong outcome | (33) |
| Karaca (2017) | Duplication | (34) |
| Katchi (2014) | Wrong publication type (abstract only) | (35) |
| Katchi (2014) | Duplication | (36) |
| Lacki (2024) | Wrong population | (37) |
| Lewis (1988) | Wrong publication type (abstract only) | (38) |
| Lewis (1988) | Duplication) | (39) |
| Lewis (1989) | Duplication | (40) |
| Likourezos (2014) | Wrong publication type (protocol) | (41) |
| Lin (2022) | Wrong publication type (abstract only) | (42) |
| Lin (2023) | Wrong outcome | (43) |
| Liu (2024) | Wrong drug type | (44) |
| Liu (2024) | Wrong drug type | (45) |
| Lopez (2020) | Wrong publication type (abstract only) | (46) |
| Maier (1982) | Wrong publication type | (47) |
| Maier (1983) | Wrong publication type (abstract only) | (48) |
| Maier (1983) | Wrong publication type (abstract only) | (49) |
| McGrath (2021) | Wrong drug type | (50) |
| Medeiros (2021) | Wrong drug type | (51) |
| Menichelli (2024) | Wrong drug type | (52) |
| Mert (2018) | Wrong population type (editorial) | (53) |
| Meyer (2023) | Wrong population | (54) |
| Michelson (1986) | Wrong publication type | (55) |
| Mieure (2011) | Wrong publication type (abstract only) | (56) |
| Mooss (2000) | Wrong publication type (abstract only) | (57) |
| Moskowitz (2017) | Wrong population | (58) |
| Neuss (1981) | Wrong publication type | (59) |
| Nicholson (2020) | Wrong drug type | (60) |
| Noble (2023) | Wrong publication type (abstract only) | (61) |
| Nunez (2021) | Wrong drug type | (62) |
| Ozgeyik (2021) | Wrong population type (editorial) | (63) |
| Personett (2014) | Wrong population | (64) |
| Platia (1989) | Wrong drug type | (65) |
| Ray (2024) | Wrong drug type | (66) |
| Romiti (2023) | Wrong study design | (67) |
| Sakamoto (2012) | Wrong population | (68) |
| Sandberg (2015) | Wrong outcome | (69) |
| Shen (2010) | Wrong publication type | (70) |
| Sticherling (2002) | Wrong population | (71) |
| Sun (2020) | Wrong publication type (abstract only) | (72) |
| Tamaki (2005) | Wrong publication type | (73) |
| Tanigawa (1984) | Wrong publication type | (74) |
| Tieleman (2019) | Wrong publication type (protocol) | (75) |
| Tieleman (2024) | Wrong publication type (protocol) | (76) |
| Ulimoen (2022) | Wrong publication type (protocol) | (77) |
| Ulimoen (2014) | Duplication | (78) |
| Ulimoen (2014) | Duplication | (79) |
| Slawson (2024) | Wrong publication type (editorial) | (80) |
| Waclawski (1987) | Wrong publication type | (81) |
| Walkey (2021) | Wrong publication type (protocol) | (82) |
| Wong (2024) | Wrong drug type | (83) |
| Xiao (2022) | Wrong drug type | (84) |

Table S6

| **Study (first author, year)** | **Country** | **Inclusion criteria** | **Number of patients: n** | **Sex % female** | **Mean or median age** | **Type of AF** | **Heart failure** | **Funding** |
| --- | --- | --- | --- | --- | --- | --- | --- | --- |
| Wang et al. (1980) | England | Chronic AF, previous treatment for rate control | CCA: 8  BB: 8 | CCA: 37%  BB: 37% | CCA: 73 (63-85)  BB: 73 (63-85) | Permanent (100%) | Not specified | Sandoz Products Ltd |
| Myers et al. (1987) | United states of America | Chronic AF, NYHA 1-2, normal LVEF, Left atrial enlargement | CCA: 9  BB: 9 | CCA: 0%  BB: 0% | CCA: 64 (58-66)  BB: 64 (58-66) | Permanent (100%) | Not specified | Not specified |
| James et al. (1989) | England (United Kingdom) | Chronic symptomatic AF, treated with digoxin | CCA: 12  BB: 12 | CCA: 67%  BB: 67% | CCA: 58 (44-77)  BB: 58 (44-77) | Permanent (100%) | Not specified | Not specified |
| Matsuda et al. (1991) | Japan | Chronic, isolated AF | CCA: 8  BB: 8 | CCA: 0%  BB:0% | CCA: 60 (±9)  BB: 60 (±9) | Permanent (100%) | CCA: 0%  BB:0% | Not specified |
| Dahlstrom et al. (1992) | Sweden | Chronic AF (>6 months), digoxin therapy, males (30-75years) and post-menopausal females | CCA: 8  BB:8 | CCA: 31%  BB: 31% | CCA: 60.6 (± 11.7)  BB: 60.6 (± 11.7 | Permanent (100%) | Not specified | KABI-Pharmacia, Swedish Heart Lung Foundation, and the Gothenburg  Medical Faculty. |
| Koh et al. (1995) | Korea | Chronic AF (>1 month) | CCA:35  BB:35 | CCA: 40%  BB: 40% | CCA: 52 (±2)  BB: 52 (±2) | Permanent (100%) | Total population: 11% | Inha University |
| Farshi et al. (1999) | United states of America | Chronic AF (>1 year), resistent to attempted cardioversion | CCA: 8  BB:12 | CCA: 8%  BB: 8% | CCA: 52 (±6)  BB: 52 (±6) | Permanent (100%) | Not specified | None reported |
| Olshansky et al. (2004) | United states of America | All AF, Age >65, likely to be reccurent, likely to cause illness or death, eligible, in the rate control arm. | CCA: 631  BB:77 | CCA: 44%  BB: 41% | CCA: 70 (±9)  BB: 69 (±9) | Not specified | Total population:23% | Supported by the National Heart, Lung, and Blood Institute. |
| Tsuneda et al. (2006) | Japan | Permanent AF with resting heart rate 60-80bpm using digitalis for more than 6 months | CCA: 22  BB:19 | CCA: 16%  BB: 9% | CCA: 66 (±8)  BB: 69 (±8) | Permanent (100%) | CCA: 14%  BB:11% | supported by Chugai Pharmaceutical Co Ltd |
| Climent et al. (2010) | Spain | Persistent AF | CCA: 3  BB:3 | Not specified | Not specified | Persistent (100%) | Not specified | Not specified |
| Ulimoen et al. (2013) | Norway | Permanent AF (>3 months), resting heart rate ≥ 80bpm or average heart rate ≥ 100bpm | CCA:60  BB:60 | CCA: 30%  BB: 30% | CCA: 71 (±9)  BB: 71 (±9) | Permanent (100%) | Total population: 0% | South-Eastern Norway Regional Health  Authority Medical Research Foundation, Bærum Hospital, Roche diagnostics |
| Scheuermeyer et al. (2013) | Canada | Primary diagnosis of AF on the emergency department | CCA: 100  BB:159 | CCA: 45%  BB: 43% | CCA: 67 (±15)  BB: 67 (±15) | CCA: <48h: (25%), >48h: (75%)  BB: <48h: (28%) , >48h: (72%) | CCA: 9%  BB: 9% | The authors have disclosed no conflicts of interest |
| Ulimoen et al. (2014) | Norway | Permanent AF (>3 months), resting heart rate ≥ 80bpm or average heart rate ≥ 100bpm | CCA: 60  BB:60 | CCA: 30%  BB: 30%: | CCA: 71 (±9)  BB: 71 (±9) | Permanent (100%) | otal population: 0% | South-Eastern Norway Regional Health  Authority Medical Research Foundation, Bærum Hospital, Roche diagnostics |
| Corino et al. (2015, feb) | Norway | Permanent AF (>3 months), resting heart rate ≥ 80bpm or average heart rate ≥ 100bpm | CCA: 60  BB:60 | CCA: 30%  BB: 30% | CCA: 71 (±9)  BB: 71 (±9) | Permanent (100%) | otal population: 0% | South-Eastern Norway Regional Health  Authority Medical Research Foundation, Bærum Hospital, Roche diagnostics |
| Horjen et al. (2016) | Norway | Permanent AF (>3 months), resting heart rate ≥ 80bpm or average heart rate ≥ 100bpm | CCA: 60  BB:60 | CCA: 30%  BB: 30% | CCA: 71 (±9)  BB: 71 (±9) | Permanent (100%) | otal population: 0% | South-Eastern Norway Regional Health  Authority Medical Research Foundation, Bærum Hospital, Roche diagnostics |
| Yu et al. (2018) | Korea | AF (1 admisison with AF or 2 outpatient visits with AF), age ≥20 | CCA: 331  BB:1037 | CCA: 48%  BB: 42% | Without HF: 64 ± 11, with HF: 71 ± 10  Not specified per rate control group | Not specified | Total population 65% | National Research Foundation of Korea |
| You et al. (2018) | Korea | AF (1 admisison with AF or 2 outpatient visits with AF), single rate control drug, asthma or COPD disease treated by bronchodilator or steroid therapy | CCA: 2482  Selective BB: 2378  Nonselective BB: 2255 | CCA: 44%  Selective BB: 47%  Nonselective BB: 49% | CCA: 74 (±9)  Selective BB: 73 (±10)  Nonselective BB: 72 (±11) | Not specified | CCA: 37% Selective BB: 50%  Nonselective BB: 53% | Not specified |
| Zaman et al. (2021) | United states of America | Age >65, AF, likely to be reccurent, likely to cause illness or death, eligible for rate and rhyhtm contol therapy, in the rate control arm treated with a single rate control drug | CCA: 344  BB:485 | CCA: 41%  BB: 39% | CCA: 70 (±8)  BB: 69 (±8) | Not specified | CCA: 15%  BB: 16% | Department of Medicine at the Pennsylvania State College of  Medicine |
| Koldenhof et al. (2022) | Netherlands | First detected AF, paroxysmal AF treated with either beta blockers or verapamil | CCA: 47  BB:383 | CCA: 51%  BB:39% | CCA: 60 (55-68)  BB: 66 (59-72) | Paroxysmal (100%) | CCA: 1%  BB: 9% | Netherlands healthcare insurance  companies (DSW, ACHMEA, and CZ), Boehringer Ingelheim, Bayer,  Pfizer, Bristol-Myers Squibb, and Daiichi Sankyo |
| Sherf et al. (2022) | Israel | Hospitalized with new onset AF | CCA: 143  BB:163 | CCA: 64%  BB: 64% | CCA: 66 (±14)  BB: 67 (±13) | New onset (100%) | Not specified | Not specified |
| Barcia et al. (2024) | Spain | All patients with AF | CCA: 2558  BB:246 | CCA: 63%  BB: 57% | CCA: 82 (±5)  BB: 80 (±5) | CCA: Permanent (73%)  BB: permanent: 77% | CCA: 7%  BB: 8% | Not specified |
| Koldenhof et al. (2023) | United states of America and Canada | Non-permanent AF, Age >65, likely to be reccurent, likely to cause illness or death, eligible | CCA: 218  BB:256 | CCA: 44%  BB: 41% | CCA: 70 (±8)  BB: 68 (±8) | Paroxysmal or persistent AF (100%) | CCA: 18%  BB: 23% | Reported no funding |
| Menichelli et al. (2024) | Italy | Using oral anticoagulation | CCA: 1246  BB:273 | Total population 46%, not specified per group | Not specified per group total population 75 (±9) | No specified | Not specified per group total population 14% | Reported no funding |
| Enge et al. (2024) | Norway | Adults symptomatic, permanent AF ≥ 3 months; resting heart rate ≥ 80 bpm; no ongoing rate-controlling treatment at inclusion; preserved left ventricular systolic function. | CCA: 49  BB:44 | CCA: 24%  BB:36% | CCA: 71 (±6)  BB: 72 (±7) | Permanent (100%) | Not specified, heart failure with reduced ejection fraction excluded | South-Eastern Norway Regional Health Authority and Vestre Viken Hospital Trust |

Abbreviations: AF: atrial fibrillation, CCA: calcium channel antagonist, BB: beta blocker,

Table S7: The Clinical Diversity In Meta-analyses (CDIM) tool

|  |  | Mean 24 hour heart rate | Maximum heart rate |
| --- | --- | --- | --- |
| Setting | 1. Setting | 1 | 1 |
| Population | 2. Age | 1 | 1 |
|  | 3. Sex | 1 | 1 |
|  | 4. Participant inclusion criteria and baseline disease severity | 0 | 0 |
|  | 5. Comorbidities | 1 | 1 |
| Intervention | 6. Intensity, strengths, or duration of intervention | 2 | 2 |
|  | 7. Timing | 0 | 1 |
|  | 8. Control intervention | 0 | 0 |
|  | 9. Cointerventions | 0 | 0 |
|  | 10. Definition of the outcome in the meta-analysis | 0 | 0 |
|  | 11. Timing of outcome measurement | 0 | 0 |
| Total score |  | 6 | 7 |

Table S8: sensitivity analysis of mean 24hour heart rate:

| Study | Mean difference | SE | P-value | lower | upper | I2 | lower.I2 | upper.I2 |
| --- | --- | --- | --- | --- | --- | --- | --- | --- |
| Omitting Wang et al. (1980) | 3,041 | 3,511 | 0,386 | -3,841 | 9,923 | 0,925 | 0,840 | 0,965 |
| Omitting Koh et al. (1995) | 1,012 | 3,523 | 0,774 | -5,892 | 7,916 | 0,600 | 0,000 | 0,867 |
| Omitting Farshi et al. (1999) | 3,809 | 4,017 | 0,343 | -4,064 | 11,682 | 0,925 | 0,840 | 0,965 |
| Omitting Tsuneda et al. (2006) | 3,374 | 4,189 | 0,420 | -4,835 | 11,584 | 0,925 | 0,841 | 0,965 |
| Omitting Ulimoen at al. (2013) | 8,416 | 1,238 | <0,001 | 5,991 | 10,842 | 0,000 | 0,000 | 0,847 |
|  |  |  |  |  |  |  |  |  |
| Pooled estimate | 3,626 | 3,313 | 0,274 | -2,867 | 10,119 | 0,901 | 0,797 | 0,951 |

Table S9: sensitivity analysis of maximum heart rate during exercise:

| Study | Mean difference | SE | P-value | lower 95% border | upper 95% border | I2 | lower.I2 | upper.I2 |
| --- | --- | --- | --- | --- | --- | --- | --- | --- |
| Omitting Myers et al. (1987) | 9,354 | 3,884 | 0,016 | 1,742 | 16,966 | 0,721 | 0,397 | 0,871 |
| Omitting Matsuda et al. (1991) | 11,250 | 3,848 | 0,003 | 3,708 | 18,792 | 0,700 | 0,342 | 0,863 |
| Omitting Dahlstrom et al. (1992) | 11,490 | 3,901 | 0,003 | 3,844 | 19,137 | 0,686 | 0,306 | 0,858 |
| Omitting Koh et al. (1995) | 6,090 | 2,992 | 0,042 | 0,227 | 11,953 | 0,000 | 0,000 | 0,708 |
| Omitting Farshi et al. (1999) | 9,925 | 3,878 | 0,010 | 2,325 | 17,525 | 0,725 | 0,408 | 0,873 |
| Omitting Tsuneda et al. (2006) | 9,889 | 4,091 | 0,016 | 1,870 | 17,907 | 0,725 | 0,407 | 0,873 |
| Omitting Ulimoen at al. (2013) | 12,396 | 3,889 | 0,001 | 4,773 | 20,018 | 0,557 | 0,000 | 0,810 |
| Omitting Enge (2024) | 11,977 | 4,055 | 0,003 | 4,030 | 19,924 | 0,600 | 0,083 | 0,826 |
|  |  |  |  |  |  |  |  |  |
| Pooled estimate | 10,524 | 3,681 | 0,004 | 3,309 | 17,738 | 0,680 | 0,328 | 0,848 |
| studlab | MD | SE | P-value | lower 95% border | upper 95% border | I2 | lower.I2 | upper.I2 |

Figure S1: sensitivity analysis using the restricted maximum likelihood model


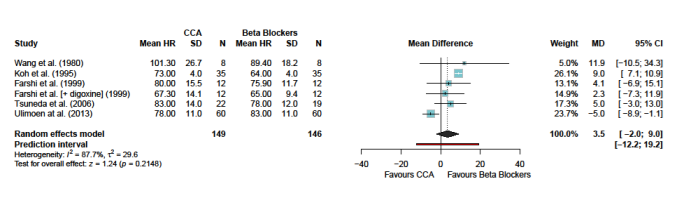


## References

1. Ahuja RC, Sinha N, Saran RK, Jain AK, Hasan M. Digoxin or verapamil or metoprolol for heart rate control in patients with mitral stenosis - a randomised cross-over study. Int J Cardiol. 1989;25(3):325–31.

2. Atzema CL, Austin PC. Rate Control With Beta-blockers Versus Calcium Channel Blockers in the Emergency Setting: Predictors of Medication Class Choice and Associated Hospitalization. Acad Emerg Med. 2017;24(11):1334–48.

3. Balser JR, Martinez EA, Winters BD, Perdue PW, Clarke AW, Huang W, et al. Beta-adrenergic blockade accelerates conversion of postoperative supraventricular tachyarrhythmias. Anesthesiology. 1998;89(5):1052‐1059.

4. Balser JR, Martinez EA, Winters BD, Perdue PW, Clarke AW, Huang W, et al. β-adrenergic blockade accelerates conversion of postoperative supraventricular tachyarrhythmias. Anesthesiology. 1998;89(5):1052–9.

5. Bedford JP, Johnson A, Redfern O, Gerry S, Doidge J, Harrison D, et al. Comparative effectiveness of common treatments for new-onset atrial fibrillation within the ICU: Accounting for physiological status. J Crit Care. 2022;67:149–56.

6. Bertaglia E, D’Este D, Zerbo F, Michieletto M, Pascotto P. Effects of verapamil and metoprolol on recovery from atrial electrical remodelling after electrical cardioversion of long-lasting atrial fibrillation: relation to immediate and early recurrences. Eur Heart J. 2001;22:561.

7. Bertaglia E, D’Este D, Zerbo F, Michieletto M, Pascotto P. Effects of verapamil and metoprolol on recovery from atrial electrical remodeling after cardioversion of long-lasting atrial fibrillation. Int J Cardiol. 2003;87(2):167–72.

8. Bosch NA, Rucci JM, Massaro JM, Winter MR, Quinn EK, Chon KH, et al. Comparative Effectiveness of Heart Rate Control Medications for the Treatment of Sepsis-Associated Atrial Fibrillation. Chest. 2021;159(4):1452–9.

9. Botto, De Nittis GL. Persistent atrial fibrillation: a cross-over randomized study on the pharmacological modulation of ventricular frequency with once-a-day adminsitration of diltiazem or metoprolol. G Ital Cardiol. 1999;29:125.

10. NCT02025465. Randomized Trial Comparing Diltiazem and Metoprolol For Atrial Fibrillation Rate Control. https://clinicaltrials.gov/show/NCT02025465. 2013;

11. Chen CW, Massera D, Aneke C, Pina I. Intravenous Diltiazem, as Compared to Intravenous Metoprolol, for Atrial Fibrillation/Flutter Is Associated with Increased Admission from the Emergency Department. J Am Coll Cardiol. 2016;67(13):786.

12. Compagner CT, Wysocki CR, Reich EK, Zimmerman LH, Holzhausen JM. Intravenous metoprolol versus diltiazem for atrial fibrillation with concomitant heart failure. Am J Emerg Med. 2022;62:49–54.

13. Corino VD, Platonov PG, Enger S, Tveit A, Ulimoen SR. Circadian variation of variability and irregularity of heart rate in patients with permanent atrial fibrillation: relation to symptoms and rate control drugs. Am J Physiol Hear Circ Physiol. 2015;309(12):H2152-7.

14. Demircan C, Cikriklar HI, Engindeniz Z, Cebicci H, Atar N, Guler V, et al. Comparison of the effectiveness of intravenous diltiazem and metoprolol in the management of rapid ventricular rate in atrial fibrillation. Emerg Med J. 2005;22(6):411–4.

15. Demir MC, Doğan M, Polat E, Akpinar G. Intravenous diltiazem or metoprolol administration in the emergency department for acute rate control of atrial fibrillation patients with rapid ventricular response with unknown ejection fraction. Duzce Med J. 2021;23(1):10–4.

16. Demircan C, Cikriklar HI, Engindeniz Z, Cebicci H, Atar N, Guler V, et al. Comparison of the effectiveness of intravenous diltiazem and metoprolol in the management of rapid ventricular rate in atrial fibrillation. Emerg Med J. 2005 Jun;22(6):411–4.

17. Desai VC, Kelton CM, Metzger AH, Cavanaugh TM, Guo JJ, Heaton PC. Comparative persistence on β-blockers versus calcium channel blockers for ventricular rate control in nonelderly patients with atrial fibrillation. Ann Pharmacother. 2014;48(12):1570–9.

18. Feeney ME, Rowe SLB, Mah ND, Barton CA, Ran R. Achieving ventricular rate control in patients taking chronic beta-blocker therapy. Am J Emerg Med. 2018;36(1):110–3.

19. Frick W, Zhang Z, Rogers L, Rojulpote C, Lin CJ. Practice patterns of rate control in atrial fibrillation and clinical outcomes from a nationwide cohort. Vol. 49, Current Problems in Cardiology. Elsevier Inc.; 2024.

20. Friedman HS, Nguyen T, Sterman H, Melniker L. Comparative Effects of Ouabain, Propranolol, and Verapamil on Myocardial Blood-Flow and Energetics during Atrial-Fibrillation. Clin Res. 1986;34(2):A300–A300.

21. Fromm C, Suau SJ, Cohen V, Likourezos A, Jellinek-Cohen S, Rose J, et al. Diltiazem vs. Metoprolol in the management of atrial fibrillation or flutter with rapid ventricular rate in the emergency department. J Emerg Med. 2015;49(2):175–82.

22. Gloaguen A. Metoprolol versus diltiazem in the acute management of atrial fibrillation in patients with heart failure with impaired ejection fraction. Ann Fr Med D Urgence. 2019;9(2):132.

23. Hargrove KL, Robinson EE, Lusk KA, Hughes DW, Neff LA, Fowler AL. Comparison of sustained rate control in atrial fibrillation with rapid ventricular rate: Metoprolol vs. Diltiazem. Am J Emerg Med. 2021;40:15–9.

24. Harvey S, Wilson K, Brad Hall A. Diltiazem versus esmolol for acute rate control in the emergency department. Am J Emerg Med. 2017;35(11):1760–2.

25. Hasbrouck M, Nguyen TT. Acute management of atrial fibrillation in congestive heart failure with reduced ejection fraction in the emergency department. Am J Emerg Med. 2022;58:39–42.

26. Hassan S, Ahmad S, Kamalakannan D, Khoury R, Kakish E, Maria V, et al. Conversion of atrial fibrillation to sinus rhythm during treatment with intravenous esmolol or diltiazem: A prospective, randomized comparison. J Cardiovasc Pharmacol Ther. 2007;12(3):227–31.

27. Hilleman DE, Reyes AP, Mooss AN, Packard KA. Esmolol versus diltiazem in atrial fibrillation following coronary artery bypass graft surgery. Curr Med Res Opin. 2003;19(5):376–82.

28. Hines MC, Reed BN, Ivaturi V, Bontempo LJ, Bond MC, Hayes BD. Diltiazem versus metoprolol for rate control in atrial fibrillation with rapid ventricular response in the emergency department. Am J Heal Pharm. 2016;73(24):2068–76.

29. Hirschy R, Ackerbauer K, DeMott J, Peksa G, O’Donnell P. Metoprolol Versus Diltiazem: Acute Management of Atrial Fibrillation in Patients with Heart Failure. Crit Care Med. 2018;46(1):87.

30. Hirschy R, Ackerbauer KA, Peksa GD, O’Donnell EP, DeMott JM. Metoprolol vs. diltiazem in the acute management of atrial fibrillation in patients with heart failure with reduced ejection fraction. Am J Emerg Med. 2019;37(1):80–4.

31. Kanorskii SG, Zingilevskii KB. Comparative effects of amiodarone, diltiazem, and atenolol on results of electrical cardioversion in patients with chronic atrial fibrillation. Kardiologiia. 2002;42(8):44–5.

32. Kapustova K, Phan B, Allison-Aipa T, Choi M. Acute rate control with metoprolol versus diltiazem in atrial fibrillation with heart failure with reduced ejection fraction. Am J Emerg Med. 2023;67:126–9.

33. Karaca I, Coşkun N, Yavuzkir M, Iikay E, Daǧli N, Işik A, et al. Effect of diltiazen and metoprolol on left atrial appendix functions in patients with nonvalvular chronic atrial fibrillation. Anadolu Kardiyol Derg. 2007;7(1):37–41.

34. Karaca I, Coşkun N, Yavuzkir M, Ilkay E, Dağli N, Işik A, et al. Effect of diltiazem and metoprolol on left atrial appendix functions in patients with nonvalvular chronic atrial fibrillation. Anadolu Kardiyol Derg [Anatolian J Cardiol. 2007;7(1):37‐41.

35. Katchi F, Nagabandi S, Shuster J, Novak E, Joseph S. Treating Rapid Atrial Fibrillation in Decompensated Heart Failure: Metoprolol Is Superior to Diltiazem. Cardiology. 2014;128:466.

36. Katchi F, Nagabandi S, Shuster J, Novak E, Joseph S. Treating Rapid Atrial Fibrillation in Acute Decompensated Heart Failure: Metoprolol and Diltiazem are Equally Safe, yet Metoprolol Increases Conversion to Sinus Rhythm. J Card Fail. 2014;20(8):S41–S41.

37. Lacki A, Martinez-Millana A. A Comparison of the Impact of Pharmacological Treatments on Cardioversion, Rate Control, and Mortality in Data-Driven Atrial Fibrillation Phenotypes in Critical Care. Bioengineering. 2024 Mar 1;11(3).

38. Lewis R V, McMurray J, McDevitt DG. The Effects of Atenolol, Verapamil and Xamoterol Upon Heart-Rate and Exercise Tolerance in Digitalized Patients with Atrial-Fibrillation. Scott Med J. 1988;33(2):251.

39. Lewis R V, McMurray J, McDevitt DG. Effects of Verapamil, Atenolol and Xamoterol in Digitalized Patients with Atrial-Fibrillation. Br J Clin Pharmacol. 1988;26(2):P213–4.

40. Lewis R V, McMurray J, McDevitt DG. Effects of atenolol, verapamil, and xamoterol on heart rate and exercise tolerance in digitalised patients with chronic atrial fibrillation. J Cardiovasc Pharmacol. 1989;13(1):1–6.

41. NCT01914926. Comparison of Diltiazem and Metoprolol in the Management of Acute Atrial Fibrillation or Atrial Flutter. https://clinicaltrials.gov/show/NCT01914926.

42. Lin SJ, Chen NY, Chang YC, Cheng CL. Comparative safety of beta-blocker and calcium channel blockers in patients with chronic obstructive pulmonary disease and atrial fibrillation: A national population cohort study in Taiwan. Pharmacoepidemiol Drug Saf. 2022;31:272.

43. Lin SJ, Liao XM, Chen NY, Chang YC, Cheng CL. Beta-blockers reduce severe exacerbation in patients with mild chronic obstructive pulmonary disease with atrial fibrillation: A population-based cohort study. BMJ Open Respir Res. 2023 Nov 20;10(1).

44. Liu S, Stiell I, Eagles D, Borgundvaag B, Grewal K. Hypotension and respiratory events related to electrical cardioversion for atrial fibrillation or atrial flutter in the emergency department. Can J Emerg Med. 2024 Feb 1;26(2):103–10.

45. Liu Y, Liu J, Wang D. The relationship between different ventricular rate control levels and cardiac remodeling in early persistent atrial fibrillation: a prospective cohort study. Front Cardiovasc Med. 2024;11.

46. Lopez B, Maloney R. Evaluation of Metoprolol Versus Diltiazem for Treatment of Atrial Fibrillation in Critical Care Unit. Crit Care Med. 2020;48.

47. Maier WD, Gigler G, Bilgin Y, Neuss H, Schlepper M. Differential Effect of Hemodynamics in Tachycardial Atrial-Fibrillation through Metoprolol and Verapamil. Z Kardiol. 1982;71(3):184.

48. Maier WD, Neuss H, Bilgin Y, Gigler G, Thormann J, Schlepper M. Modification of hemodynamics in tachycardiac atrial fibrillation by metoprolol and verapamil. Z Kardiol. 1983;72(8):465‐470.

49. Maier WD, Neuss H, Bilgin Y. Influence of metoprolol and verapamil on hemodynamics in atrial fibrillation with fast ventricular response. Z Kardiol. 1983;72(8):465–70.

50. McGrath P, Kersten B, Chilbert MR, Rusch C, Nadler M. Evaluation of metoprolol versus diltiazem for rate control of atrial fibrillation in the emergency department. Am J Emerg Med. 2021;46:585–90.

51. Medeiros T, Bui V, Almekdash MH, Keesari R, Lee YR. Rate control with intravenous diltiazem, verapamil, and metoprolol in acute atrial fibrillation with rapid ventricular rate. SAGE Open Med. 2021 Jan 25;9:205031212110177.

52. Menichelli D, Poli D, Antonucci E, Palareti G, Pignatelli P, Pastori D. Renin-angiotensin-aldosterone system inhibitors and mortality risk in elderly patients with atrial fibrillation. Insights from the nationwide START registry. Eur J Intern Med. 2024 Jan 1;119:84–92.

53. Mert G, Dural M, Mert KU. Comparison of acute management of atrial fibrillation in heart failure with unequal sized groups. Am J Emerg Med. 2018;36(9):1693.

54. Meyer M, Wetmore JB, Weinhandl ED, Roetker NS. Association of Nondihydropyridine Calcium Channel Blockers Versus β-Adrenergic Receptor Blockers With Risk of Heart Failure Hospitalization. Am J Cardiol. 2023 Jun 15;197:68–74.

55. Michelson EL, Porterfield JK, Das G, Platia E V, Sawin HS, Macvaugh H, et al. A Comparison of Esmolol and Verapamil in the Treatment of Atrial-Fibrillation Flutter. J Am Coll Cardiol. 1986;7(2):A157–A157.

56. Mieure KD, Moranville MP, Park JJ, Lat I, Jennings HR, Lazar S, et al. A comparison of intravenous rate control agents for new onset atrial fibrillation with rapid ventricular rate. Hear Rhythm. 2011;8(5):S216‐S217.

57. Mooss AN, Wurdeman RL, Mohiuddin SM, Reyes AP, Sugimoto JT, Scott W, et al. Esmolol versus diltiazem in the treatment of postoperative atrial fibrillation/atrial flutter after open heart surgery. Am Heart J. 2000;140(1):176–80.

58. Moskowitz A, Chen KP, Cooper AZ, Chahin A, Ghassemi MM, Celi LA. Management of atrial fibrillation with rapid ventricular response in the intensive care unit: A secondary analysis of electronic health record data. Shock. 2017;48(4):436–40.

59. Neuss H, Schlepper M. Beta receptor blockers versus calcium-antagonists in the control of heart rate in chronic atrial fibrillation. Eur Heart J. 1981;2:38.

60. Nicholson J, Czosnowski Q, Flack T, Pang PS, Billups K. Hemodynamic comparison of intravenous push diltiazem versus metoprolol for atrial fibrillation rate control. Am J Emerg Med. 2020;38(9):1879–83.

61. Noble M. Diltiazem Versus Metoprolol for Atrial Fibrillation with Rapid Ventricular Rate in the Ed. Crit Care Med. 2023;51(1):72.

62. Nuñez Cruz S, DeMott JM, Peksa GD, Slocum GW. Evaluation of the blood pressure effects of diltiazem versus metoprolol in the acute treatment of atrial fibrillation with rapid ventricular rate. Am J Emerg Med. 2021;46:329–34.

63. Ozgeyik M, Ozgeyik MO. Hemodynamic comparison of intravenous push diltiazem versus metoprolol for atrial fibrillation rate control. Am J Emerg Med. 2021;45:654.

64. Personett HA, Smoot DL, Stollings JL, Sawyer M, Oyen LJ. Intravenous Metoprolol Versus Diltiazem for Rate Control in Noncardiac, Nonthoracic Postoperative Atrial Fibrillation. Ann Pharmacother. 2014;48(3):314–9.

65. Platia E V., Michelson EL, Porterfield JK, Das G. Esmolol versus verapamil in the acute treatment of atrial fibrillation or atrial flutter. Am J Cardiol. 1989 Apr 15;63(13):925–9.

66. Ray WA, Chung CP, Stein CM, Smalley W, Zimmerman E, Dupont WD, et al. Serious Bleeding in Patients with Atrial Fibrillation Using Diltiazem with Apixaban or Rivaroxaban. JAMA. 2024 May 14;331(18):1565–75.

67. Romiti GF, Corica B, Mei DA, Frost F, Bisson A, Boriani G, et al. Impact of chronic obstructive pulmonary disease in patients with atrial fibrillation: An analysis from the GLORIA-AF registry. Europace. 2024 Jan 1;26(1).

68. Sakamoto A, Kitakaze M, Takamoto S, Namiki A, Kasanuki H, Hosoda S. Landiolol, an ultra-short-acting β₁-blocker, more effectively terminates atrial fibrillation than diltiazem after open heart surgery: prospective, multicenter, randomized, open-label study (JL-KNIGHT study). Circ J. 2012;76(5):1097‐1101.

69. Sandberg F, Corino VD, Mainardi LT, Ulimoen SR, Enger S, Tveit A, et al. Non-invasive assessment of the effect of beta blockers and calcium channel blockers on the AV node during permanent atrial fibrillation. J Electrocardiol. 2015;48(5):861‐866.

70. Shen SL, Zhao YC. A comparative study on the efficacy and safety of intravenous esmolol, amiodarone and diltiazem for controlling rapid ventricular rate of patients with atrial fibrillation during anesthesia period. Zhonghua Xin Xue Guan Bing Za Zhi. 2010;38(11):989–92.

71. Sticherling C, Tada H, Hsu W, Bares AC, Oral H, Pelosi F, et al. Effects of diltiazem and esmolol on cycle length and spontaneous conversion of atrial fibrillation. J Cardiovasc Pharmacol Ther. 2002;7(2):81–8.

72. Sun E, Diab K, Leyton C, Nnani D, Murthy S, Patricia C, et al. Acute Rate Control of Atrial Fibrillation with Rapid Ventricular Response Using Intravenous Calcium Channel Blockers or Intravenous Beta Blockers Leads to Cardiogenic Shock in Patients with Underlying Systolic Dysfunction. J Am Coll Cardiol. 2020;75(11):844.

73. Tamaki S, Yamada T, Mine T, Morita T, Kioka H, Tsukamoto Y, et al. The beneficial effects of beta-blocker and calcium-channel blocker on atrial fibrillation in patients with chronic heart failure. J Card Fail. 2005;11(9):S295–S295.

74. Tanigawa M, Hirata M, Kimura M, Matsumoto Y, Kitano K, Kiya F, et al. Effects of Digoxin, Diltiazem and Propranolol on the Heart-Rate in Chronic Atrial-Fibrillation. Japanese Circ Journal-English Ed. 1984;48(8):861–2.

75. NTR6745. EN: which drug prevents worsening of paroxysmal atrial fibrillation: verapamil or metoprolol? NL: welk medicijn voorkomt het hardnekkiger worden van paroxismaal atriumfibrilleren: verapamil of metoprolol? https://trialsearch.who.int/Trial2.aspx?TrialID=NTR6745.

76. NL-OMON44431. Effects of VERApamil versus metoprolol to prevent progression from Paroxysmal to persistent Atrial Fibrillation. https://trialsearch.who.int/Trial2.aspx?TrialID=NL-OMON44431.

77. NCT02695992. Rate Control in Atrial Fibrillation II. https://clinicaltrials.gov/show/NCT02695992.

78. Ulimoen SR, Enger S, Norseth J, Pripp AH, Abdelnoor M, Arnesen H, et al. Improved rate control reduces cardiac troponin T levels in permanent atrial fibrillation. Clin Cardiol. 2014;37(7):422–7.

79. Ulimoen SR, Enger S, Pripp AH, Abdelnoor M, Arnesen H, Gjesdal K, et al. Calcium channel blockers improve exercise capacity and reduce N-terminal Pro-B-type natriuretic peptide levels compared with beta-blockers in patients with permanent atrial fibrillation. Eur Heart J. 2014 Feb 21;35(8):517–23.

80. Slawson DC. Greater Risk of Serious Bleeding With Diltiazem vs. Metoprolol in Adults With Atrial Fibrillation Using Apixaban or Rivaroxaban. Am Fam Physician. 2024;110(2):203.

81. Waclawski SH, Pluth TA, Brooks S. Management of Acute-Onset Atrial-Fibrillation Flutter - Esmolol Vs Verapamil Vs Digoxin Vs Placebo Ev Platia. Circulation. 1987;76(4):520.

82. NCT04234477. Assessment of Intravenous Rate Control Response in Atrial Fibrillation Trial (AIRCRAFT). https://clinicaltrials.gov/show/NCT04234477.

83. Wong AYS, Warren-Gash C, Bhaskaran K, Leyrat C, Banerjee A, Smeeth L, et al. Potential interactions between medications for rate control and direct oral anticoagulants: Population-based cohort and case-crossover study. Hear Rhythm. 2024 Dec 1;21(12):2445–54.

84. Xiao SQ, Ibarra F, Cruz M. Intravenous Metoprolol Versus Diltiazem for Rate Control in Atrial Fibrillation. Ann Pharmacother. 2022;56(8):916–21.
